# Tissue-specific changes in *Srebf1* and *Srebf2* expression and DNA methylation with perinatal phthalate exposure

**DOI:** 10.1093/eep/dvz009

**Published:** 2019-06-20

**Authors:** Laura Moody, Diego Hernández-Saavedra, Daniel G Kougias, Hong Chen, Janice M Juraska, Yuan-Xiang Pan

**Affiliations:** 1 Division of Nutritional Sciences, University of Illinois at Urbana-Champaign, Urbana, IL, USA; 2 Neuroscience Program, University of Illinois at Urbana-Champaign, Urbana, IL, USA; 3 Department of Food Science and Human Nutrition, University of Illinois at Urbana-Champaign, Urbana, IL, USA; 4 Department of Psychology, University of Illinois at Urbana-Champaign, Urbana, IL, USA

**Keywords:** gene expression, lipid metabolism, transcription, hypermethylation, epigenetic mechanisms

## Abstract

Perinatal exposure to endocrine disrupting chemicals negatively impacts health, but the mechanism by which such toxicants damage long-term reproductive and metabolic function is unknown. Lipid metabolism plays a pivotal role in steroid hormone synthesis as well as energy utilization and storage; thus, aberrant lipid regulation may contribute to phthalate-driven health impairments. In order to test this hypothesis, we specifically examined epigenetic disruptions in lipid metabolism pathways after perinatal phthalate exposure. During gestation and lactation, pregnant Long–Evans rat dams were fed environmentally relevant doses of phthalate mixture: 0 (CON), 200 (LO), or 1000 (HI) µg/kg body weight/day. On PND90, male offspring in the LO and HI groups had higher body weights than CON rats. Gene expression of lipid metabolism pathways was altered in testis and adipose tissue of males exposed to the HI phthalate dosage. Specifically, *Srebf1* was downregulated in testis and *Srebf2* was upregulated in adipose tissue. In testis of HI rats, DNA methylation was increased at two loci and reduced at one other site surrounding *Srebf1* transcription start site. In adipose tissue of HI rats, we observed increased DNA methylation at one region within the first intron of *Srebf2*. Computational analysis revealed several potential transcriptional regulator binding sites, suggesting functional relevance of the identified differentially methylated CpGs. Overall, we show that perinatal phthalate exposure affects lipid metabolism gene expression in a tissue-specific manner possibly through altering DNA methylation of *Srebf1* and *Srebf2*.

## Introduction

Phthalates are environmental toxicants that have endocrine disrupting effects. Phthalates can be found in many household plastic and cosmetic products [1, 2] and as contaminants in foods such as poultry and dairy [3]. Due to phthalate influence on hormone balance, epidemiological studies have extensively examined the relationship between phthalates and male reproductive health. Phthalate exposure in adulthood has been associated with reduced sperm quality and testosterone levels [4–6], while prenatal phthalate exposure results in shorter anogenital distance in infants [7]. Several animal models have confirmed and expanded upon these observations. Early-life exposure has been of particular interest due to heightened sensitivity during the critical period and the potential to impact long-term health outcomes. In addition to validating findings in human studies [8, 9], rodent models have shown that perinatal phthalate exposure disrupts Leydig [10, 11] and Sertoli [12, 13] cell populations as well as induces aberrant DNA methylation [14, 15] and histone modifications [10, 16] in the testis.

Additionally, studies have quantified metabolic outcomes after phthalate exposure. While some have suggested a positive association between early-life phthalate exposure and body mass index (BMI) [17, 18], others have found no correlation [19] or a negative correlation between perinatal phthalates, BMI, waist circumference, and body fat percentage [20, 21]. Similar inconsistencies have been reported in animal models. Indeed, early-life phthalate exposure in rodents has been shown to have positive [22, 23], negative [24–26], and no relationship [27, 28] with body weight. There remains no clear consensus as to whether early-life phthalate exposure produces sustained metabolic complications. Thus, understanding the possible mechanisms by which phthalates affect reproduction and metabolism is critical in clarifying the long-term health implications of environmental exposure.

Proper metabolic and reproductive function involves appropriate regulation of lipid synthesis, catabolism, and storage. Obesity is characterized by excessive fat storage and hyperlipidemia. As for reproductive health, not only does adiposity impact endocrine function, but cholesterol itself is also a precursor of androgen and estrogen. Previous experimentation in animal and *in vitro* models suggests that phthalates may induce lipid accumulation by upregulating expression of fatty acid synthesis and lipid transport genes [28–34]. Such metabolic processes are highly regulated and involve a number of transcription factors. Given their widespread regulatory role in lipid metabolism, sterol regulatory element binding proteins (SREBPs) have been hypothesized to underlie phthalate-induced metabolic dysregulation [35, 36]. When cellular sterol levels are low, SREBP cleavage-activating protein (SCAP) escorts SREBP from the endoplasmic reticulum to the Golgi apparatus, where it is cleaved and released by Site-1 protease and Site-2 protease [37]. SREBP then translocates to the nucleus and can bind sterol response elements to induce transcription of genes such as fatty acid synthase (*Fasn*), acetyl CoA carboxylase (*Acc*), and HMG-CoA synthase (*Hmgcs*) [38, 39]. SREBP1 upregulates lipogenesis and triglyceride synthesis while SREBP2 promotes cholesterol uptake and synthesis. Thus, SREBPs are critical in responding to dietary conditions and maintaining metabolic health across tissues.

We sought to uncover the impact of perinatal phthalate exposure on lipid metabolism in key metabolic and reproductive tissues. We hypothesized that aberrant DNA methylation and elevated expression of SREBP genes may underlie the metabolic and reproductive abnormalities caused by phthalates. In the current experiment, we asked whether perinatal phthalate exposure impacts lipid metabolism in adult offspring. Long–Evans rat dams consumed environmentally relevant doses of phthalates during gestation and lactation. Male offspring were sacrificed at postnatal day (PND) 90 and gene expression and DNA methylation were measured in testis, adipose, and liver. Overall we show that phthalate exposure drives tissue-specific changes in gene expression and potentially operates through an epigenetic mechanism.

## Results

### Body Weight and Composition

**Figure 1:**
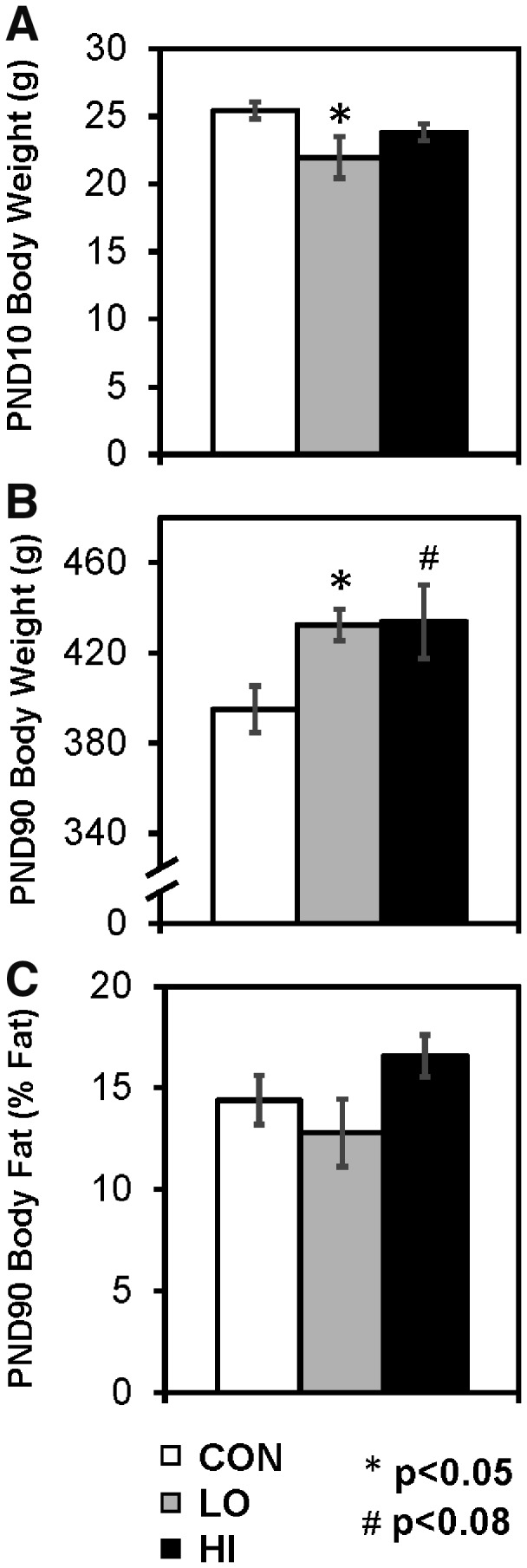
body weights for male offspring on (**A**) PND10 and (**B**) PND90. (**C**) Body fat percentage on PND90. Values are represented as mean ± SEM. *P < 0.05, #*P* < 0.08

Pregnant Long–Evans rat dams were fed one of three phthalate dosages from gestational day 2 until PND10: 0 [control (CON)], 200 [low (LO)], or 1000 [high (HI)] µg/kg of body weight/day. Body weights of male offspring were measured at PND10 and 90. At PND10, the LO group had significantly lower body weights than controls (*P* = 0.015; Fig. 1A). However, at PND90, this trend was reversed, as both LO and HI animals had higher body weights than control (*P* = 0.0080 and *P* = 0.070, respectively; Fig 1B). Additionally, body fat and lean mass were measured on PND90. LO rats had slightly lower and HI rats had slightly higher average body fat percentage than CON, however neither of these were significant (*P* = 0.29 and *P* = 0.31, respectively; Fig. 1C).

### Gene Expression

On PND90, animals were sacrificed and testis, gonadal adipose, and liver were removed for analysis. Due to the endocrine disrupting properties of phthalates and the important role of lipids in hormone synthesis, we measured genes involved in tissue-specific as well as general lipid metabolic processes. In testis, we measured cholesterol and hormone synthesis, lipogenesis, and transcriptional regulation genes. In phthalate-exposed animals, we found a decrease in Niemann–Pick intracellular cholesterol transporter 1 (*Npc1*; *P* = 0.042), fatty acid synthase (*Fasn*; *P* = 0.012), and *Srebf1* (*P* = 0.027; Table 1). These differences were only observed in the HI group. Moreover, we saw a slight decrease in glycerol-3-phosphate acyltransferase (*Gpam*; *P* = 0.076) and stearoyl-CoA desaturase (*Scd*; *P* = 0.084). In the LO group, there was a non-significant increase in *Srebf2* expression (*P* = 0.065).

**Table 1:** gene expression in testis at PND90 (values are represented as mean ± SEM)

Gene	CON	LO	HI
Cholesterol and hormone synthesis			
Cyp11a1	0.56 (0.17)	0.60 (0.13)	0.44 (0.021)
Cyp19a1	1.29 (0.24)	1.52 (0.22)	1.15 (0.11)
Hmgcr	1.08 (0.16)	1.50 (0.17)	1.2 (0.077)
Hsd17b3	0.51 (0.054)	0.70 (0.10)	0.54 (0.04)
Insig1	1.10 (0.041)	1.17 (0.11)	1.00 (0.047)
Npc1	0.99 (0.16)	0.84 (0.097)	0.64 (0.034)*
Scap	0.87 (0.16)	0.92 (0.14)	0.68 (0.032)
Scp2	1.04 (0.054)	1.06 (0.11)	1.07 (0.051)
Star	0.56 (0.099)	0.57 (0.082)	0.57 (0.052)
Tspo	0.80 (0.16)	0.81 (0.13)	0.57 (0.031)
Fatty acids and triacylglycerols synthesis			
Acacb	1.03 (0.10)	1.19 (0.081)	1.01 (0.052)
Fasn	0.82 (0.059)	0.78 (0.073)	0.66 (0.025)*
Gpam	0.96 (0.18)	0.76 (0.061)	0.64 (0.016)**
Scd	0.89 (0.17)	0.78 (0.084)	0.60 (0.014)
Transcriptional regulators			
Lxra	0.97 (0.045)	1.05 (0.14)	0.91 (0.042)
Srebf1	0.77 (0.088)	0.72 (0.047)	0.56 (0.015)*
Srebf2	0.94 (0.053)	1.18 (0.10)**	0.92 (0.031)

* *P* < 0.05.

** *P* < 0.08.

In adipose tissue, we measured an increase in *Fasn* (*P* = 0.040) and *Srebf2* (*P* = 0.031; Table 2). We found slight increases in expression of cholesterol-regulating gene, insulin induced gene 1 (*Insig1*; *P* = 0.061) as well as *Scd* (*P* = 0.073). In liver, only minimal gene expression differences were observed (Table 3). *Scd* was upregulated in HI group, but this was not statistically significant (*P* = 0.066).

**Table 2:** gene expression in gonadal adipose at PND90 (values are represented as mean ± SEM)

Gene	CON	LO	HI
Cholesterol			
Hmgcr	1.14 (0.12)	1.11 (0.12)	1.18 (0.13)
Insig1	1.51 (0.22)	2.07 (0.48)	2.86 (0.58)**
Npc1	1.50 (0.12)	1.21 (0.15)	1.49 (0.28)
Scap	1.19 (0.13)	1.21 (0.20)	1.38 (0.20)
Fatty acids and triacylglycerols synthesis			
Acacb	2.13 (0.41)	1.51 (0.29)	1.92 (0.37)
Fasn	0.57 (0.12)	0.73 (0.19)	1.87 (0.53)*
Gpam	1.32 (0.20)	1.13 (0.19)	1.69 (0.26)
Scd	1.30 (0.42)	1.21 (0.33)	4.34 (1.27)**
Transcriptional regulators			
Srebf1	1.32 (0.25)	1.46 (0.24)	1.42 (0.20)
Srebf2	0.90 (0.12)	1.23 (0.15)	1.61 (0.20)*
Lxra	1.93 (0.36)	1.89 (0.37)	1.96 (0.30)

* *P* < 0.05.

** *P* < 0.08.

**Table 3:** gene expression in liver at PND90 (values are represented as mean ± SEM)

Gene	CON	LO	HI
Lipid import			
Ldlr	1.22 (0.23)	1.25 (0.16)	1.67 (0.33)
Scarb1	0.75 (0.070)	0.79 (0.065)	0.81 (0.11)
Lipid export			
Mttp	1.13 (0.089)	1.08 (0.055)	1.12 (0.14)
ApoB	0.84 (0.056)	0.76 (0.064)	0.85 (0.13)
ApoE	1.44 (0.20)	1.07 (0.20)	1.09 (0.16)
Cholesterol			
Hmgcr	1.40 (0.20)	1.06 (0.21)	1.50 (0.37)
Insig1	1.27 (0.35)	1.02 (0.12)	1.44 (0.29)
Npc1	0.86 (0.16)	0.75 (0.083)	0.76 (0.10)
Scap	0.78 (0.049)	0.88 (0.071)	0.92 (0.092)
Fatty acids and triacylglycerols synthesis			
Acacb	1.10 (0.27)	1.44 (0.27)	1.42 (0.29)
Fasn	0.73 (0.10)	1.00 (0.33)	1.28 (0.34)
Gpam	0.64 (0.077)	0.78 (0.15)	0.73 (0.15)
Scd	1.03 (0.31)	1.61 (0.53)	2.61 (0.70)**
Transcriptional regulators			
Lxra	1.44 (0.17)	1.50 (0.13)	1.73 (0.26)
Srebf1	0.76 (0.17)	1.02 (0.22)	1.06 (0.18)
Srebf2	0.82 (0.049)	0.87 (0.075)	0.87 (0.064)

* *P* < 0.05.

** *P* < 0.08.

### DNA Methylation

**Figure 2:**
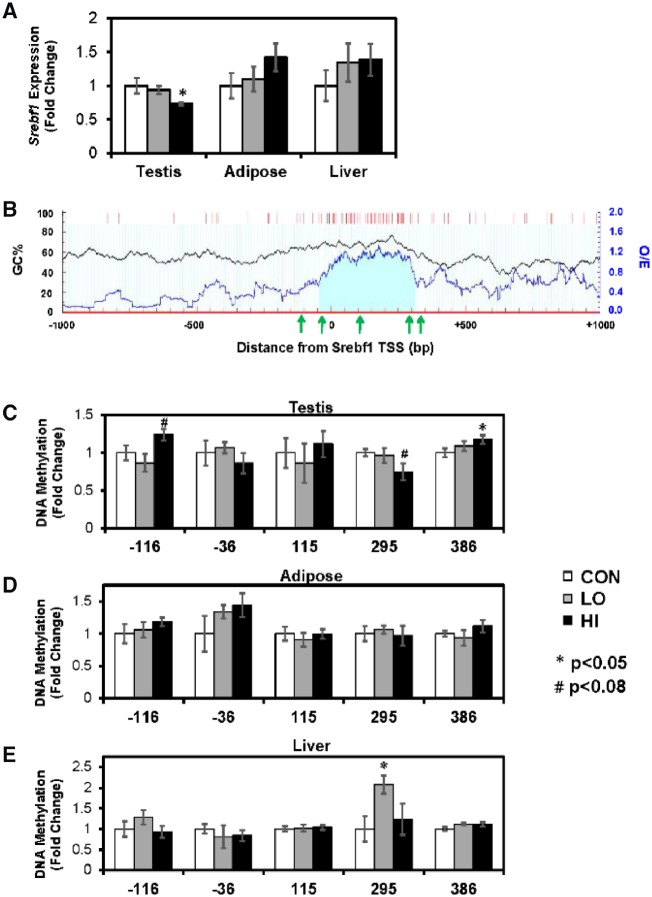
(**A**) mRNA expression of *Srebf1* across tissues. (**B**) CpG distribution around the *Srebf1* transcription start site (TSS). Black line graph represents the guanine-cytosine content across the region (GC%). Blue line graph represents observed/expected CpG ratio (observed/expected). Red bars represent CpG sites. Light blue shaded area represents CpG island. Green arrows represent location of CpGs measured by MSP. Bottom three graphs show DNA methylation in (**C**) testis, (**D**) adipose, and (**E**) liver. Gene expression and DNA methylation values are represented as mean fold change compared to CON ± SEM. *P < 0.05, #*P* < 0.08

Next, we tested the hypothesis that differences in gene expression may be caused by phthalate-mediated alterations in DNA methylation. We identified several differentially expressed lipid metabolism genes, but due to their potential for widespread transcriptional regulation, we only measured DNA methylation associated with *Srebf1* and *Srebf2*. MSP was performed for several sites surrounding the transcription start site (TSS) of the two genes. The HI phthalate had reduced testicular *Srebf1* expression, whereas in adipose and liver, *Srebf1* expression was unchanged (Fig. 2A). The sequence of *Srebf1* includes one cytosine phosphate guanine dinucleotides (CpG) island straddling the TSS (Fig. 2B). We measured DNA methylation of three CpG loci located in islands and one locus in either shore. In testis, we detected increased DNA methylation in the HI animals within the upstream and downstream shores (*P* = 0.061 and *P* = 0.048, respectively; Fig. 2C). Additionally, there was a decrease in methylation in the HI group in the island CpGs located 295 bp downstream of the TSS (*P* = 0.080). There were not DNA methylation differences in adipose tissue (Fig. 2D). In liver, there was increased DNA methylation in the LO group in the island CpGs 295 bp downstream of the TSS (*P* = 0.011; Fig. 2E).

**Figure 3:**
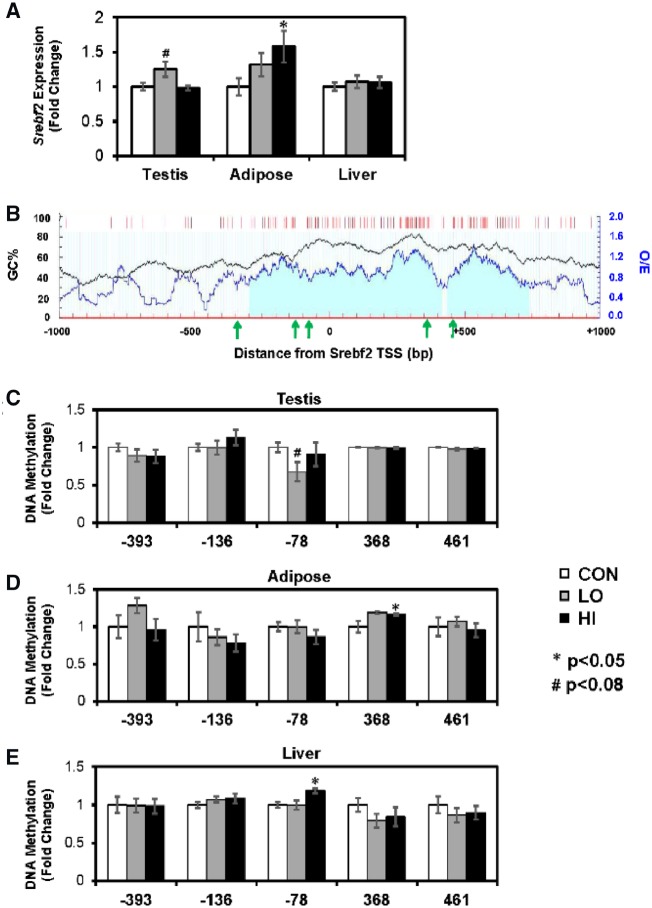
(**A**) mRNA expression of *Srebf2* across tissues. (**B**) CpG distribution around the *Srebf2* transcription start site (TSS). Black line graph represents the guanine-cytosine content across the region (GC%). Blue line graph represents observed/expected CpG ratio (observed/expected). Red bars represent CpG sites. Light blue shaded area represents CpG island. Green arrows represent location of CpGs measured by MSP. Bottom three graphs show DNA methylation in (**C**) testis, (**D**) adipose, and (**E**) liver. Gene expression and DNA methylation values are represented as mean fold change compared to CON ± SEM. *P < 0.05, #*P* < 0.08

DNA methylation changes around *Srebf2* were minimal. Gene expression in adipose tissue was significantly increased in the HI group. In testis, there was a slight increase in the LO group (Fig. 3A). Surrounding the *Srebf2* TSS, there is one large CpG island (Fig. 3B). Another CpG island is located ∼500 bp downstream. In testis, there was a non-significant decrease in DNA methylation in the LO group at CpGs located 78 bp upstream of the TSS (*P* = 0.058; Fig. 3A). The same locus was differentially methylated in the liver, where it was hypermethylated in the HI group (*P* = 0.0021; Fig. 3E). In adipose, there was increased DNA methylation at an intronic site located 368 bp downstream of the TSS and within the CpG island (*P* = 0.048; Fig. 3D).

### Transcription Factor Prediction

**Figure 4:**
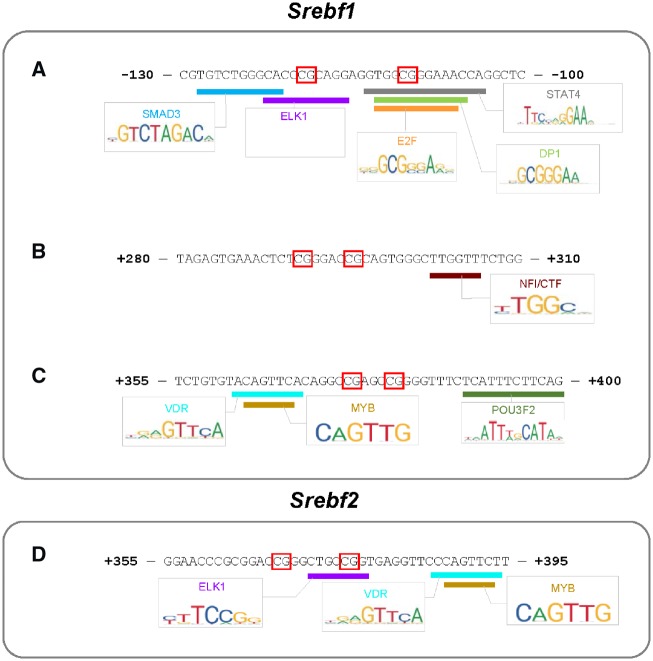
computationally predicted transcription factor binding sites at differentially methylated loci around (**A**, **B**, **C**) *Srebf1* and (**D**) *Srebf2*. Red boxes represent differentially methylated CpGs as measured by MSP in testis (*Srebf1*) and adipose (*Srebf2*)

Finally, we attempted to predict whether the identified differentially methylated regions might impact transcription and expression of *Srebf1* and *Srebf2*. We performed a bioinformatics analysis of the differentially methylated regions using PROMO. First, we looked at three regions around *Srebf1* that were differentially methylated in the testis. Within the site 130–100 bp upstream of *Srebf1*, we found potential binding sites for SMAD3, ELK1, E2F, DP1, and STAT4 (Fig. 4A). Within the region 280–310 bp downstream of the Srebf1 TSS, there was a potential binding site for nuclear factor I/CAAT box transcription factor (NFI/CTF; Fig. 4B). Within the region 355–400 bp downstream of the Srebf1 TSS, we identified putative binding sites for VDR, MYB, and POU3F2 (Fig. 4C). In Srebf2, we examined the region 355–395 bp downstream of the TSS and found sites for ELK1, VDR, and MYB binding (Fig. 4D).

## Discussion

In this paper, we have demonstrated the long-lasting effect of endocrine disruptors on lipid metabolism. During the perinatal period, Long–Evans rats were administered a mixture of phthalates at concentrations observed in human populations. Phthalates altered early postnatal and adolescent body weight but did not alter fat deposition. Gene expression analysis at PND90 revealed minor alterations in hepatic lipid metabolism genes, but significant changes in cholesterol and fatty acid synthesis in gonadal adipose tissue. Finally, high doses of perinatal phthalates significantly altered cholesterol trafficking and fatty acid metabolism genes in testis. In particular the transcriptional regulator *Srebf1* was downregulated, possibly through hypermethylation of its promoter and coding region. Early-life phthalate exposure might interfere with adipose and testicular fatty acid and cholesterol synthesis through epigenetic mechanisms, and thus alter reproductive function.

### Phthalates and Lipid Metabolism

**Figure 5:**
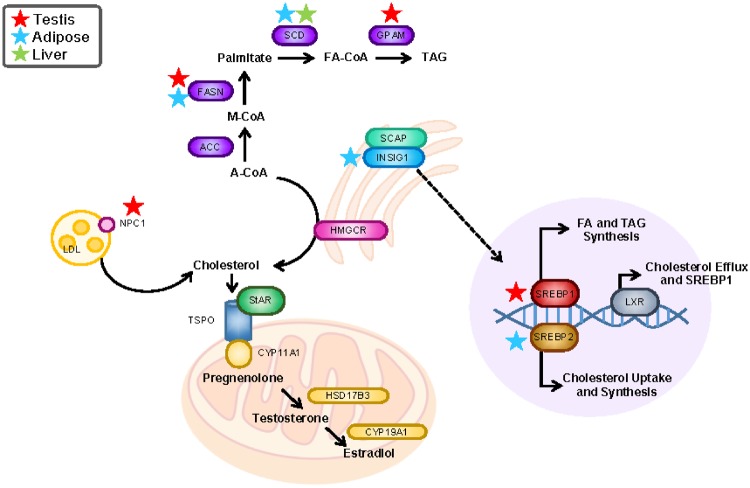
summary of the effects of perinatal phthalate exposure on lipid metabolism pathways. Gene expression changes in different tissue types are noted with stars (*P* < 0.08)

Lipid metabolism is an integral part of reproductive and general metabolic health. Through highly regulated pathways, fatty acids, triglycerides, and cholesterol are synthesized in response to environmental and homeostatic stimuli in order to meet metabolic demands (Fig. 5). Such processes are transcriptionally regulated by factors including SREBP1, which upregulates fatty acid and triglyceride synthesis; SREBP2, which promotes cholesterol uptake and synthesis; and LXR, which opposes the effects of SREBP2 and activates SREBP1 (Fig. 5). In testis, cholesterol plays a particularly critical role as a precursor of testosterone (Fig. 5).

Previous investigation has suggested that exposure to individual phthalates impacts lipid metabolism. In *Caenorhabditis elegans*, life-long exposure to phthalates DEHP and DEP increased fat accumulation along with survival and reproductive function, possibly related to the upregulation of fatty acid synthesis (*fasn-1, pod-2, fat-5, elo-2, acs-6, sbp-1*) and lipid transport genes (*vit-2, -4, -5,* and *-6*) [29]. In zebrafish, water-exposure to diisononyl phthalate (DiNP) activated orexigenic signals in the brain and steatohepatitis in adult animals [30]. In human cells, exposure to monoethylhexyl phthalate (MEHP) affected adipocyte size, possibly via increased lipolysis, glucose uptake, and oxygen consumption [31]. In placental cells MEHP produced lipidome-wide changes with specific alterations in glycerolipids and glycerophospholipids and promoted triacylglycerols accumulation [34]. Interestingly, prenatal DEHP exposure in rats was shown to target adrenal gland peroxisome proliferator-activated receptor (PPAR) and cholesterol biosynthesis pathways [33], which rendered offspring more susceptibility to second-hit stressor challenge [32]. Similarly, *in utero* DEHP exposure has been shown to potentiate the effect of a postnatal high fat-diet [28]. In our study, we used a phthalate mixture to mimic human environmental exposure. However just as individual phthalates induce transcriptional changes in lipid metabolism genes in different tissues, we also observed specific adipose tissue and testicular gene expression changes in cholesterol and fatty acid synthesis pathways.

Lipid metabolism genes that are affected by phthalates vary among tissues, and depend on the window of exposure. In our paper, prenatal exposure to low or high doses of phthalates produced no significant effects in liver. This finding contradicts previous studies that have shown phthalate-mediated changes in hepatic morphology and *Ppara* gene expression [40, 41]. This difference might be due to the low phthalate dose used in our study (1000× lower). Furthermore, the lack of gene expression changes in liver might not be reflective of the ability of the liver to handle fatty acids and cholesterol, given that the animals were exposed to neither a high fat-diet nor a second stressor later in life.

In adipose tissue, we showed a significant increase in cholesterol, fatty acid, and lipid metabolism-related genes after phthalate treatment. A trend toward increased *Insig1* expression might speak to altered cholesterol signaling, which is vital for the production of hormones within adipose tissue. Importantly, *Srebf2* (Srebp2) was significantly upregulated by a high dose of phthalates, suggesting higher cholesterol synthesis as its main target genes include *Hmgcr* and *Hmgcs*, key enzymes for cholesterol biosynthesis [37, 42]. Moreover, increases in *Fasn* and *Scd* indicate a prominent effect on lipid synthesis with high phthalate doses, possibly affecting the ability of adipocytes to accumulate fat.

Lastly, a high dosage of perinatal phthalates decreased expression of genes involved with cholesterol, fatty acid, and triglyceride metabolism, namely *Npc1*, *Fasn*, *Gpam*, and *Srebf1*. Dysregulation of *Srebf1* (Srepb1) is known to affect both fatty acid and cholesterol metabolism via the two known isoforms of SREBP protein 1a and 1c [43]. Indeed, *Npc1*, *Fasn*, *Gpam* are transcriptional targets of Srebp1 [38, 44, 45]. This suggests that the phthalate-induced decrease in expression of these genes may be a result of decreased SREBP1 binding. Previous studies have shown that exposure to DiBP (600 mg/kg BW/day) caused dysregulation of testicular fatty acid and cholesterol-metabolism genes, which impacts steroidogenesis and reproductive function [41]. Studies have shown that androgen production does not require the accumulation of SREBP2 protein [46], thus highlighting the effect of *Srebf1* in regulating fatty acid and cholesterol metabolism, and in turn modulating the hormone production of testis and reproductive function.

### Phthalates and DNA Methylation

In this study, we provide evidence that early-life phthalate exposure induces different DNA methylation patterns around the *Srebf1* and *Srebf2* promoters. Several others have highlighted the epigenetic implications of phthalates. In rat testis, maternal phthalate exposure resulted in hypermethylation of steroidogenic factor-1 (SF-1) and specific protein-1 (Sp-1) [14] and hypomethylation of the mineralocorticoid receptor [47]. Phthalates also induced decreased global DNA hydroxymethylation [48] and increased global DNA methylation [49, 50]. In adipose tissue, we have previously observed decreased methylation in the adipogenesis gene, frizzled 1 (*Fzd1*), and the triglyceride cleaving enzyme, lipoprotein lipase (*Lpl*) [51]. Phthalate administration in rodent models also resulted in hepatic hypomethylation of the oncogene, c-Myc [52, 53], while another study reported no change in global DNA methylation [54]. Our results add to the literature by demonstrating that phthalates alter DNA methylation in a tissue-specific manner.

Our findings support the idea that DNA methylation in the promoter prevents transcription factor binding and induces a closed chromatin confirmation in order to repress transcription. We found three regions around the *Srebf1* TSS that were differentially methylated in the testis. Two of the three were located in CpG shores and were hypermethylated after phthalate exposure. This finding is consistent with previous literature showing that CpG shores are more vulnerable to differential methylation than CpG islands [55, 56]. Additionally, *Srebf1* gene expression was downregulated in testis; thus, an increase in DNA methylation follows the canonical assumption that DNA methylation blocks transcription. Transcription factor analysis identified SMAD3, ELK1, E2F, DP1, and STAT4 binding sites within the upstream shore (130–100 bp upstream of *Srebf1* TSS). Each of these factors has been shown to activate transcription [57–61]. Within the downstream shore (355–4000 bp downstream of the *Srebf1* TSS), there was a potential binding site for VDR, MYB, and POU3F2, which have also been demonstrated to have activational properties [62–64].

We identified two differentially methylated CpG loci that showed a positive association between DNA methylation and gene expression. First, in testis we observed decreased DNA methylation at the CpG site 295 bp downstream of the Srebf1 TSS. This decrease was associated with decreased gene expression. Upon examination of the transcription factor binding sites, we saw that relatively few factors bind to the region. NFI/CTF may activate or repress transcription depending on recruitment of other cofactors and chromatin modifiers [65]. However, the same locus was highly methylated in the liver, despite no change in gene expression. Thus, it may be the case that DNA methylation at this site is not critical for the transcriptional regulation of *Srebf1*. Secondly, in adipose tissue we observed increased *Srebf2* expression accompanied by increased DNA methylation at the region 355–395 bp downstream of the TSS. Binding sites for ELK1, VDR, and MYB were found. Although much of the literature points to the activating role of these transcription factors, there is evidence that ELK1 and MYB display repressive characteristics in certain contexts [66–68]. It is possible that increased methylation could increase transcription by inhibiting repressor binding.

## Conclusion

Here we demonstrate the tissue-specific implications of perinatal phthalate exposure. We are among the first to investigate phthalate-induced DNA methylation across tissues; however, our experiments should be viewed in light of a few limitations. First, our sample size was relatively small (*n* = 8–9/group). This might be one reason that we found several genes and CpGs that were slightly but not statistically altered with phthalate treatment. Our data can be used to generate hypotheses and should be replicated in a larger cohort. Secondly, while we measured body weight and body composition as metabolic outcomes, we did not quantify reproductive outcomes beyond changes in gene expression. Future experimentation should focus on discovering associations between DNA methylation at the identified loci and physiological outcomes such as sperm count and quality as well as other fertility measures. Additionally, we only looked at a limited number of CpG sites using MSP. To gain a more comprehensive picture of phthalate-mediated DNA methylation, genome-wide methods such as bisulfite sequencing or methylated DNA immunoprecipitation sequencing (MeDIP-seq) may be utilized. Finally, future experiments can confirm the impact of DNA methylation and TF binding within the identified loci. We measured differential methylation at CpGs within the *Srebf1* and *Srebf2* promoters, but it is unclear whether these differences directly impact transcription or whether they are consequences of gene expression changes. Moreover, we report several computationally predicted TF binding sites around *Srebf1* and *Srebf2*, however these findings should be validated for transcriptional relevance through appropriate *in vitro* assays.

Overall, we show that perinatal phthalate treatment induces distinct gene expression changes in lipid metabolism pathways in testis and adipose tissue but not in the liver. Importantly, expression and DNA methylation of *Srebf1* and *Srebf2* were altered by phthalates. We propose that perinatal phthalate exposure alters DNA methylation around lipid-related transcription factors, changes the expression of their gene targets, and alters lipid metabolism. This study provides insight into the mechanism by which phthalates might disrupt reproductive and general metabolic outcomes.

## Methods

### Animals

Three-month old Long–Evans hooded female rats (*n* = 8–9 per group) were obtained from Harlan Laboratories (Indianapolis, IN). Before breeding, rats were housed in same-sex pairs and were given *ad libitum* access to a low phytoestrogen diet (Harlan 2020X; Taklad Diets, Madison, WI). During breeding, dams were placed in suspended wire-bottom cages, and once a sperm plug was detected, dams were removed and individually housed. From gestational day 2 until PND10, dams received one of three phthalate treatments, 0 (CON), 200 (LO), or 1000 µg/kg body weight/day (HI).

The phthalate mixture was designed to reflect exposure levels found in pregnant women in Champaign—Urbana, Illinois (unpublished data) as well as exposure levels in the general US population [69]. The mixture consisted of ∼35% diethyl (DEP), 21% bis(2-ethylhexyl) (DEHP), 15% dibutyl (DBP), 15% diisononyl (DiNP), 8% diisobutyl (DiBP), and 5% benzyl butyl (BBP) phthalate. The phthalates were suspended in tocopherol-stripped corn oil at a concentration of 0, 0.6, or 3 mg phthalates/ml for the 0, 200, or 1000 µg phthalates/kg body weight/day doses, respectively. The phthalate mixture was administered orally on top of half a cookie (Newman’s Own organic alphabet cookie, vanilla flavor). Dams were acclimatized for 2 days with half a cookie topped with tocopherol-stripped corn oil, and from gestational day 2 through PND10, dams voluntarily ate the half cookie and phthalate mixture. Additionally, dams were given *ad libitum* access a control diet (15.8% kcal fat, 63.9% kcal carbohydrate, 20.3% kcal protein; D10012G, Research Diets Inc., New Brunswick, NJ).

On PND25, one male offspring from each dam was randomly selected and moved to its own individual cage (CON: *n* = 8, LO: *n* = 9, HI: *n* = 9). Offspring continued on the D10012G diet and received no phthalates for the remaining duration of the experiment. On PND90, body composition was measured using the EchoMRI-700 Body Composition Analyzer (Echo Medical Systems, Houston, TX). The rats were then sacrificed and liver, testis, and gonadal adipose tissue was dissected out. All tissues were snap-frozen in liquid nitrogen and stored at −80°C for genetic analysis. All procedures were approved by the University of Illinois Institutional Care and Use Committee and adhere to the National Institutes of Health guidelines on the ethical use of animals.

### Gene Expression

Frozen tissue was ground in liquid nitrogen and placed in a 1.5 ml tube containing Trizol. Before loading the adipose sample into the column, the tube was spun and the lipid layer was removed. Total RNA was extracted using Direct-zol RNA MiniPrep Plus (Zymo Research, Irvine, CA) with in-column DNase I treatment to eliminate DNA contamination. cDNA synthesis and qRT-PCR were performed as previously described [70]. Primers were designed (VectorNTI software; Life Technologies, Grand Island, NY), validated for specificity (Basic Local Alignment Search Tool; BLAST; https://blast.ncbi.nlm.nih.gov/Blast.cgi), and synthesized (IDT; Coralville, IA). All mRNA primer sequences and amplification efficiencies can be found in Supplementary Table S1.

### DNA Methylation

Genomic DNA was isolated (ZR Genomic DNA Tissue MiniPrep kit; Zymo Research) and bisulfite converted (EZ DNA Methylation Gold Kit; Zymo Research). The EZ DNA Methylation Gold Kit was chosen for its high bisulfite conversion efficiency, which has repeatedly been reported to range between 99 and 100% [71–74]. Thus, we can confidently assume that methylation differences are likely not artifacts of different conversion efficiencies. To perform the conversion reaction, samples were heated to 98°C for 10 min, 64°C for 2.5 h, and held at 4°C. Samples were desulphonated and diluted to 5 ng/μl for methylation specific PCR (MSP) using Sybr Green (Quanta Biosciences, Beverly, MA). Quantitative real-time PCR was performed using the StepOnePlus Real-Time PCR System (Applied Biosystems, Foster City, CA). CT values were quantified based on a serially diluted standard curve and percent methylation was calculated by dividing quantity of methylated DNA by the sum of methylated and unmethylated DNA. Methylation was normalized to the CON group.

Methylated and unmethylated primer pairs were manually designed (Primer Express 3.0.1 software’s Primer Probe Test Tool; Life Technologies). The T_m_ for each primer was between 68 and 70°C and the CG content was between 30 and 80%. Primers were validated in the IDT OligoAnalyzer to minimize dimers and hairpin loops. Unmethylated DNA was measured using both unmethylated forward and reverse primers. Methylated DNA was measured by pairing one methylated primer with the corresponding unmethylated primer. All MSP primer sequences and amplification efficiencies can be found in Supplementary Table S2.

### Transcription Factor and Statistical Analysis

Computational prediction of transcription factor binding was performed with PROMO version 3.0.2 [75, 76]. Identified transcription factor sequences were validated with the Jaspar Database [77]. One way ANOVA followed by pairwise comparisons between CON vs LO and CON vs HI was used to determine statistical significance for body weight, body composition, gene expression, and DNA methylation. Statistical analysis was performed in R version 3.3.2.

## Supplementary Material

dvz009_Supplementary_Data

## References

[1] Koniecki D, Wang R, Moody RP, Zhu J. Phthalates in cosmetic and personal care products: concentrations and possible dermal exposure. Environ Res 2011;111:329–36. 21315328 10.1016/j.envres.2011.01.013

[2] Sathyanarayana S. Phthalates and children's health. Curr Probl Pediatr Adolesc Health Care 2008;38:34–49. 18237855 10.1016/j.cppeds.2007.11.001

[3] Serrano SE, Braun J, Trasande L, Dills R, Sathyanarayana S. Phthalates and diet: a review of the food monitoring and epidemiology data. Environ Health 2014;13:43. 24894065 10.1186/1476-069X-13-43 PMC4050989

[4] Hauser R, Meeker JD, Duty S, Silva MJ, Calafat AM. Altered semen quality in relation to urinary concentrations of phthalate monoester and oxidative metabolites. Epidemiology 2006;17:682–91. 17003688 10.1097/01.ede.0000235996.89953.d7

[5] Chang WH, Li SS, Wu MH, Pan HA, Lee CC. Phthalates might interfere with testicular function by reducing testosterone and insulin-like factor 3 levels. Hum Reprod 2015;30:2658–70. 26385792 10.1093/humrep/dev225

[6] Meeker JD, Ferguson KK. Urinary phthalate metabolites are associated with decreased serum testosterone in men, women, and children from NHANES 2011–2012. J Clin Endocrinol Metab 2014;99:4346–52. 25121464 10.1210/jc.2014-2555 PMC4223430

[7] Swan SH, Main KM, Liu F, Stewart Sl, Kruse Rl, Calafat AM, Mao CS, Redmon JB, Ternand Cl, Sullivan S et al Decrease in anogenital distance among male infants with prenatal phthalate exposure. Environ Health Perspect 2005;113:1056–61. 16079079 10.1289/ehp.8100 PMC1280349

[8] Gray LE Jr, Furr J, Tatum-Gibbs KR, Lambright C, Sampson H, Hannas BR, Wilson VS, Hotchkiss A, Foster PM. Establishing the “biological relevance” of dipentyl phthalate reductions in fetal rat testosterone production and plasma and testis testosterone levels. Toxicol Sci 2016;149:178–91. 26454885 10.1093/toxsci/kfv224 PMC4715258

[9] Li N, Chen X, Zhou X, Zhang W, Yuan J, Feng J. The mechanism underlying dibutyl phthalate induced shortened anogenital distance and hypospadias in rats. J Pediatr Surg 2015;50:2078–83. 26385564 10.1016/j.jpedsurg.2015.08.046

[10] Kilcoyne KR, Smith LB, Atanassova N, Macpherson S, McKinnell C, van den Driesche S, Jobling MS, Chambers TJ, De Gendt K, Verhoeven G et al Fetal programming of adult Leydig cell function by androgenic effects on stem/progenitor cells. Proc Natl Acad Sci USA 2014;111:E1924–32. 24753613 10.1073/pnas.1320735111 PMC4020050

[11] Wakui S, Shirai M, Motohashi M, Mutou T, Oyama N, Wempe MF, Takahashi H, Inomata T, Ikegami M, Endou H, Asari M. Effects of in utero exposure to di(n-butyl) phthalate for estrogen receptors alpha, beta, and androgen receptor of Leydig cell on rats. Toxicol Pathol 2014;42:877–87. 24067674 10.1177/0192623313502879

[12] Auharek SA, de Franca LR, McKinnell C, Jobling MS, Scott HM, Sharpe RM. Prenatal plus postnatal exposure to Di(n-Butyl) phthalate and/or flutamide markedly reduces final Sertoli cell number in the rat. Endocrinology 2010;151:2868–75. 20392824 10.1210/en.2010-0108

[13] Wang Y, Yang Q, Liu W, Yu M, Zhang Z, Cui X. Di(2-ethylhexyl) phthalate exposure in utero damages Sertoli cell differentiation via disturbance of sex determination pathway in fetal and postnatal mice. Toxicol Sci 2016;152:53–61. 27060630 10.1093/toxsci/kfw063

[14] Sekaran S, Jagadeesan A. In utero exposure to phthalate downregulates critical genes in Leydig cells of F1 male progeny. J Cell Biochem 2015;116:1466–77. 25649163 10.1002/jcb.25108

[15] Chen J, Wu S, Wen S, Shen L, Peng J, Yan C, Cao X, Zhou Y, Long C, Lin T et al The mechanism of environmental endocrine disruptors (DEHP) induces epigenetic transgenerational inheritance of cryptorchidism. PLoS One 2015;10:e0126403. 26035430 10.1371/journal.pone.0126403 PMC4452760

[16] Liu C, Qian P, Yang L, Zhang L, Chen C, He M, Lu Y, Feng W, Li M, Zhang Y et al Pubertal exposure to di-(2-ethylhexyl)-phthalate inhibits G9a-mediated histone methylation during spermatogenesis in mice. Arch Toxicol 2016;90:955–69. 25975992 10.1007/s00204-015-1529-2

[17] Buckley JP, Engel SM, Braun JM, Whyatt RM, Daniels Jl, Mendez MA, Richardson DB, Xu Y, Calafat AM, Wolff MS et al Prenatal phthalate exposures and body mass index among 4- to 7-year-old children: a pooled analysis. Epidemiology 2016;27:449–58. 26745610 10.1097/EDE.0000000000000436 PMC4821741

[18] Hatch EE, Nelson JW, Qureshi MM, Weinberg J, Moore Ll, Singer M, Webster TF. Association of urinary phthalate metabolite concentrations with body mass index and waist circumference: a cross-sectional study of NHANES data, 1999–2002. Environ Health 2008;7:27. 18522739 10.1186/1476-069X-7-27 PMC2440739

[19] Shoaff J, Papandonatos GD, Calafat AM, Ye X, Chen A, Lanphear BP, Yolton K, Braun JM. Early-life phthalate exposure and adiposity at 8 years of age. Environ Health Perspect 2017;125:097008. 28935615 10.1289/EHP1022 PMC5915197

[20] Buckley JP, Engel SM, Mendez MA, Richardson DB, Daniels Jl, Calafat AM, Wolff MS, Herring AH. Prenatal phthalate exposures and childhood fat mass in a New York city cohort. Environ Health Perspect 2016;124:507–13. 26308089 10.1289/ehp.1509788 PMC4829985

[21] Maresca MM, Hoepner LA, Hassoun A, Oberfield SE, Mooney SJ, Calafat AM, Ramirez J, Freyer G, Perera FP, Whyatt RM et al Prenatal exposure to phthalates and childhood body size in an urban cohort. Environ Health Perspect 2016;124:514–20. 26069025 10.1289/ehp.1408750 PMC4829975

[22] Hao C, Cheng X, Guo J, Xia H, Ma X. Perinatal exposure to diethyl-hexyl-phthalate induces obesity in mice. Front Biosci (Elite Ed) 2013;5:725–33. 23277027 10.2741/e653

[23] Hao C, Cheng X, Xia H, Ma X. The endocrine disruptor mono-(2-ethylhexyl) phthalate promotes adipocyte differentiation and induces obesity in mice. Biosci Rep 2012;32:619–29. 22953781 10.1042/BSR20120042 PMC3497724

[24] Jiang JR, Sun Wl, Jing YF, Liu SB, Ma Z, Hong Y, Ma L, Qin C, Liu Q, Stratton HJ et al Prenatal exposure to di-n-butyl phthalate induces anorectal malformations in male rat offspring. Toxicology 2011;290:322–6. 22027561 10.1016/j.tox.2011.10.008

[25] Wine RN, Li LH, Barnes LH, Gulati DK, Chapin RE. Reproductive toxicity of di-n-butylphthalate in a continuous breeding protocol in Sprague-Dawley rats. Environ Health Perspect 1997;105:102–7. 9074889 10.1289/ehp.97105102 PMC1469857

[26] Li M, Qiu L, Zhang Y, Hua Y, Tu S, He Y, Wen S, Wang Q, Wei G. Dose-related effect by maternal exposure to di-(2-ethylhexyl) phthalate plasticizer on inducing hypospadiac male rats. Environ Toxicol Pharmacol 2013;35:55–60. 23228707 10.1016/j.etap.2012.10.006

[27] Saillenfait AM, Gallissot F, Sabate JP, Remy A. Prenatal developmental toxicity studies on diundecyl and ditridecyl phthalates in Sprague-Dawley rats. Reprod Toxicol 2013;37:49–55. 23376823 10.1016/j.reprotox.2013.01.004

[28] Strakovsky RS, Lezmi S, Shkoda I, Flaws JA, Helferich WG, Pan YX. In utero growth restriction and catch-up adipogenesis after developmental di (2-ethylhexyl) phthalate exposure cause glucose intolerance in adult male rats following a high-fat dietary challenge. J Nutr Biochem 2015;26:1208–20. 26188368 10.1016/j.jnutbio.2015.05.012 PMC4631689

[29] Pradhan A, Olsson PE, Jass J. Di(2-ethylhexyl) phthalate and diethyl phthalate disrupt lipid metabolism, reduce fecundity and shortens lifespan of *Caenorhabditis elegans*. Chemosphere 2018;190:375–82. 29020644 10.1016/j.chemosphere.2017.09.123

[30] Forner-Piquer I, Maradonna F, Gioacchini G, Santangeli S, Allara M, Piscitelli F, Habibi HR, Di Marzo V, Carnevali O. Dose-specific effects of di-isononyl phthalate on the endocannabinoid system and on liver of female zebrafish. Endocrinology 2017;158:3462–76. 28938452 10.1210/en.2017-00458

[31] Chiang HC, Kuo YT, Shen CC, Lin YH, Wang Sl, Tsou TC. Mono(2-ethylhexyl)phthalate accumulation disturbs energy metabolism of fat cells. Arch Toxicol 2016;90:589–601. 25543134 10.1007/s00204-014-1446-9

[32] Lee S, Martinez-Arguelles DB, Campioli E, Papadopoulos V. Fetal exposure to low levels of the plasticizer DEHP predisposes the adult male adrenal gland to endocrine disruption. Endocrinology 2017;158:304–18. 27849367 10.1210/en.2016-1604

[33] Martinez-Arguelles DB, Campioli E, Lienhart C, Fan J, Culty M, Zirkin BR, Papadopoulos V. In utero exposure to the endocrine disruptor di-(2-ethylhexyl) phthalate induces long-term changes in gene expression in the adult male adrenal gland. Endocrinology 2014;155:1667–78. 24564399 10.1210/en.2013-1921

[34] Petit J, Wakx A, Gil S, Fournier T, Auzeil N, Rat P, Laprevote O. Lipidome-wide disturbances of human placental JEG-3 cells by the presence of MEHP. Biochimie 2018;149:1–8. 29567412 10.1016/j.biochi.2018.03.002

[35] Johnson KJ, McDowell EN, Viereck MP, Xia JQ. Species-specific dibutyl phthalate fetal testis endocrine disruption correlates with inhibition of SREBP2-dependent gene expression pathways. Toxicol Sci 2011;120:460–74. 21266533 10.1093/toxsci/kfr020 PMC3061485

[36] Zhang W, Shen XY, Zhang WW, Chen H, Xu WP, Wei W. The effects of di 2-ethyl hexyl phthalate (DEHP) on cellular lipid accumulation in HepG2 cells and its potential mechanisms in the molecular level. Toxicol Mech Methods 2017;27:245–52. 27996362 10.1080/15376516.2016.1273427

[37] Horton JD, Goldstein Jl, Brown MS. SREBPs: activators of the complete program of cholesterol and fatty acid synthesis in the liver. J Clin Invest 2002;109:1125–31. 11994399 10.1172/JCI15593 PMC150968

[38] Amemiya-Kudo M, Shimano H, Hasty AH, Yahagi N, Yoshikawa T, Matsuzaka T, Okazaki H, Tamura Y, Iizuka Y, Ohashi K et al Transcriptional activities of nuclear SREBP-1a, -1c, and -2 to different target promoters of lipogenic and cholesterogenic genes. J Lipid Res 2002;43:1220–35. 12177166

[39] Rome S, Lecomte V, Meugnier E, Rieusset J, Debard C, Euthine V, Vidal H, Lefai E. Microarray analyses of SREBP-1a and SREBP-1c target genes identify new regulatory pathways in muscle. Physiol Genomics 2008;34:327–37. 18559965 10.1152/physiolgenomics.90211.2008

[40] Maranghi F, Lorenzetti S, Tassinari R, Moracci G, Tassinari V, Marcoccia D, Di Virgilio A, Eusepi A, Romeo A, Magrelli A et al In utero exposure to di-(2-ethylhexyl) phthalate affects liver morphology and metabolism in post-natal CD-1 mice. Reprod Toxicol 2010;29:427–32. 20307648 10.1016/j.reprotox.2010.03.002

[41] Boberg J, Metzdorff S, Wortziger R, Axelstad M, Brokken L, Vinggaard AM, Dalgaard M, Nellemann C. Impact of diisobutyl phthalate and other PPAR agonists on steroidogenesis and plasma insulin and leptin levels in fetal rats. Toxicology 2008;250:75–81. 18602967 10.1016/j.tox.2008.05.020

[42] Brown MS, Goldstein Jl. The SREBP pathway: regulation of cholesterol metabolism by proteolysis of a membrane-bound transcription factor. Cell 1997;89:331–40. 9150132 10.1016/s0092-8674(00)80213-5

[43] Shimano H. Sterol regulatory element-binding proteins (SREBPs): transcriptional regulators of lipid synthetic genes. Prog Lipid Res 2001;40:439–52. 11591434 10.1016/s0163-7827(01)00010-8

[44] Ruiz R, Jideonwo V, Ahn M, Surendran S, Tagliabracci VS, Hou YY, Gamble A, Kerner J, Irimia-Dominguez JM, Puchowicz MA et al Sterol regulatory element-binding protein-1 (SREBP-1) is required to regulate glycogen synthesis and gluconeogenic gene expression in mouse liver. J Biol Chem 2014;289:5510–7. 24398675 10.1074/jbc.M113.541110 PMC3937627

[45] Gevry N, Schoonjans K, Guay F, Murphy BD. Cholesterol supply and SREBPs modulate transcription of the Niemann–Pick C-1 gene in steroidogenic tissues. J Lipid Res 2008;49:1024–33. 18272928 10.1194/jlr.M700554-JLR200

[46] Eacker SM, Agrawal N, Qian K, Dichek Hl, Gong EY, Lee K, Braun RE. Hormonal regulation of testicular steroid and cholesterol homeostasis. Mol Endocrinol 2008;22:623–35. 18032697 10.1210/me.2006-0534 PMC2262169

[47] Martinez-Arguelles DB, Culty M, Zirkin BR, Papadopoulos V. In utero exposure to di-(2-ethylhexyl) phthalate decreases mineralocorticoid receptor expression in the adult testis. Endocrinology 2009;150:5575–85. 19819939 10.1210/en.2009-0847 PMC2795714

[48] Abdel-Maksoud FM, Leasor KR, Butzen K, Braden TD, Akingbemi BT. Prenatal exposures of male rats to the environmental chemicals bisphenol A and di(2-ethylhexyl) phthalate impact the sexual differentiation process. Endocrinology 2015;156:4672–83. 26372177 10.1210/en.2015-1077

[49] Wu S, Zhu J, Li Y, Lin T, Gan L, Yuan X, Xiong J, Liu X, Xu M, Zhao D et al Dynamic epigenetic changes involved in testicular toxicity induced by di-2-(ethylhexyl) phthalate in mice. Basic Clin Pharmacol Toxicol 2010;106:118–23. 19912166 10.1111/j.1742-7843.2009.00483.x

[50] Wu SD, Zhu J, Li YS, Lin T, Gan LQ, Yuan XG, Xu MD, Wei GH. Dynamic effect of di-2-(ethylhexyl) phthalate on testicular toxicity: epigenetic changes and their impact on gene expression. Int J Toxicol 2010;29:193–200. 20335514 10.1177/1091581809355488

[51] Moody L, Kougias D, Jung PM, Digan I, Hong A, Gorski A, Chen H, Juraska J, Pan YX. Perinatal phthalate and high-fat diet exposure induce sex-specific changes in adipocyte size and DNA methylation. J Nutr Biochem 2019;65:15–25. 30599393 10.1016/j.jnutbio.2018.11.005 PMC6547370

[52] Kostka G, Urbanek-Olejnik K, Wiadrowska B. Di-butyl phthalate-induced hypomethylation of the c-myc gene in rat liver. Toxicol Ind Health 2010;26:407–16. 20504828 10.1177/0748233710369124

[53] Ge R, Tao L, Kramer PM, Cunningham Ml, Pereira MA. Effect of peroxisome proliferators on the methylation and protein level of the c-myc protooncogene in B6C3F1 mice liver. J Biochem Mol Toxicol 2002;16:41–7. 11857776 10.1002/jbt.10019

[54] Pogribny IP, Tryndyak VP, Boureiko A, Melnyk S, Bagnyukova TV, Montgomery B, Rusyn I. Mechanisms of peroxisome proliferator-induced DNA hypomethylation in rat liver. Mutat Res 2008;644:17–23. 18639561 10.1016/j.mrfmmm.2008.06.009 PMC2571982

[55] Irizarry RA, Wu H, Feinberg AP. A species-generalized probabilistic model-based definition of CpG islands. Mamm Genome 2009;20:674–80. 19777308 10.1007/s00335-009-9222-5 PMC2962567

[56] Doi A, Park I-H, Wen B, Murakami P, Aryee MJ, Irizarry R, Herb B, Ladd-Acosta C, Rho J, Loewer S et al Differential methylation of tissue- and cancer-specific CpG island shores distinguishes human induced pluripotent stem cells, embryonic stem cells and fibroblasts. Nat Genet 2009;41:1350–3. 19881528 10.1038/ng.471 PMC2958040

[57] Besnard A, Galan-Rodriguez B, Vanhoutte P, Caboche J. Elk-1 a transcription factor with multiple facets in the brain. Front Neurosci 2011;5:35. 21441990 10.3389/fnins.2011.00035 PMC3060702

[58] Dimova DK, Dyson NJ. The E2F transcriptional network: old acquaintances with new faces. Oncogene 2005;24:2810–26. 15838517 10.1038/sj.onc.1208612

[59] Helin K, Wu Cl, Fattaey AR, Lees JA, Dynlacht BD, Ngwu C, Harlow E. Heterodimerization of the transcription factors E2F-1 and DP-1 leads to cooperative trans-activation. Genes Dev 1993;7:1850–61. 8405995 10.1101/gad.7.10.1850

[60] Massague J, Seoane J, Wotton D. Smad transcription factors. Genes Dev 2005;19:2783–810. 16322555 10.1101/gad.1350705

[61] Horvath CM. STAT proteins and transcriptional responses to extracellular signals. Trends Biochem Sci 2000;25:496–502. 11050435 10.1016/s0968-0004(00)01624-8

[62] Pike JW, Meyer MB, Lee SM, Onal M, Benkusky NA. The vitamin D receptor: contemporary genomic approaches reveal new basic and translational insights. J Clin Invest 2017;127:1146–54. 28240603 10.1172/JCI88887 PMC5373853

[63] Ramsay RG, Gonda TJ. MYB function in normal and cancer cells. Nat Rev Cancer 2008;8:523–34. 18574464 10.1038/nrc2439

[64] Tantin D. Oct transcription factors in development and stem cells: insights and mechanisms. Development 2013;140:2857–66. 23821033 10.1242/dev.095927 PMC3699277

[65] Gronostajski RM. Roles of the NFI/CTF gene family in transcription and development. Gene 2000;249:31–45. 10831836 10.1016/s0378-1119(00)00140-2

[66] Hipp N, Symington H, Pastoret C, Caron G, Monvoisin C, Tarte K, Fest T, Delaloy C. IL-2 imprints human naive B cell fate towards plasma cell through ERK/ELK1-mediated BACH2 repression. Nat Commun 2017;8:1443. 29129929 10.1038/s41467-017-01475-7 PMC5682283

[67] Allen RD 3rd, Kim HK, Sarafova SD, Siu G. Negative regulation of CD4 gene expression by a HES-1-c-Myb complex. Mol Cell Biol 2001;21:3071–82. 11287612 10.1128/MCB.21.9.3071-3082.2001 PMC86935

[68] Pastorcic M, Das HK. Ets transcription factors ER81 and Elk1 regulate the transcription of the human presenilin 1 gene promoter. Brain Res Mol Brain Res 2003;113:57–66. 12750007 10.1016/s0169-328x(03)00090-1

[69] Phthalates NRCUCotHRo (ed). Phthalates and Cumulative Risk Assessment: The Tasks Ahead. Washington (DC): National Academies Press, 2008. 25009926

[70] Zhou D, Wang H, Cui H, Chen H, Pan YX. Early-life exposure to high-fat diet may predispose rats to gender-specific hepatic fat accumulation by programming Pepck expression. J Nutr Biochem 2015;26:433–40. 25716581 10.1016/j.jnutbio.2014.10.009

[71] Herb BR, Wolschin F, Hansen KD, Aryee MJ, Langmead B, Irizarry R, Amdam GV, Feinberg AP. Reversible switching between epigenetic states in honeybee behavioral subcastes. Nat Neurosci 2012;15:1371–3. 22983211 10.1038/nn.3218 PMC3518384

[72] Heyn H, Li N, Ferreira HJ, Moran S, Pisano DG, Gomez A, Diez J, Sanchez-Mut JV, Setien F, Carmona FJ et al Distinct DNA methylomes of newborns and centenarians. Proc Natl Acad Sci USA 2012;109:10522–7. 22689993 10.1073/pnas.1120658109 PMC3387108

[73] Holmes EE, Jung M, Meller S, Leisse A, Sailer V, Zech J, Mengdehl M, Garbe LA, Uhl B, Kristiansen G et al Performance evaluation of kits for bisulfite-conversion of DNA from tissues, cell lines, FFPE tissues, aspirates, lavages, effusions, plasma, serum, and urine. PLoS One 2014;9:e93933. 24699908 10.1371/journal.pone.0093933 PMC3974851

[74] Kint S, De Spiegelaere W, De Kesel J, Vandekerckhove L, Van Criekinge W. Evaluation of bisulfite kits for DNA methylation profiling in terms of DNA fragmentation and DNA recovery using digital PCR. PLoS One 2018;13:e0199091. 29902267 10.1371/journal.pone.0199091 PMC6002050

[75] Farre D, Roset R, Huerta M, Adsuara JE, Rosello L, Alba MM, Messeguer X. Identification of patterns in biological sequences at the ALGGEN server: PROMO and MALGEN. Nucleic Acids Res 2003;31:3651–3. 12824386 10.1093/nar/gkg605 PMC169011

[76] Messeguer X, Escudero R, Farre D, Nunez O, Martinez J, Alba MM. PROMO: detection of known transcription regulatory elements using species-tailored searches. Bioinformatics 2002;18:333–4. 11847087 10.1093/bioinformatics/18.2.333

[77] Khan A, Fornes O, Stigliani A, Gheorghe M, Castro-Mondragon JA, van der Lee R, Bessy A, Cheneby J, Kulkarni SR, Tan G et al JASPAR 2018: update of the open-access database of transcription factor binding profiles and its web framework. Nucleic Acids Res 2018;46:D260–6. 29140473 10.1093/nar/gkx1126 PMC5753243

