# Supplementary material for: Tissue-specific changes in Srebf1 and Srebf2 expression and DNA methylation with perinatal phthalate exposure
Source: Environ Epigenet. 2019 Jun 20;5(2):dvz009. doi: 10.1093/eep/dvz009 (PMC6586200; doi:10.1093/eep/dvz009)
Supplement: dvz009_Supplementary_Data [file dvz009_supplementary_data.docx]

| **Supplementary Table 1. Primers for qPCR** | | |  |  |
| --- | --- | --- | --- | --- |
| Gene Name | Ensembl ID | Position | Sequence | Amplification Efficiency* |
| Acacb | ENSRNOT00000078868.1 | Forward +1081 | 5'-ACCCCAAACTTCCAGAGC -3' | 105.25% |
|  |  | Reverse +1189 | 5'-TGGGCTACAATGGTGGAG-3' |  |
| Apob | ENSRNOT00000046811 | Forward +2667 | 5'-GCATCATCATCCCAGACTTCGCTA-3' | 96.31% |
|  |  | Reverse +2734 | 5'-GCCTGACTCGTGGAAGAAGTTTGTA-3' |  |
| Apoe | ENSRNOT00000041891 | Forward +43 | 5'-GGAGGCTAAGGAGTTGTT-3' | 110.07% |
|  |  | Reverse +109 | 5'-ACAGAGCCTTCATCTTCC-3' |  |
| Cyp11a1 | ENSRNOT00000010831 | Forward +563 | 5'-TTACACAGACGCATCAAGCAGC-3' | 97.05% |
|  |  | Reverse +638 | 5'-AGGCAAAGCGGAATAGGTCATC-3' |  |
| Cyp19a1 | ENSRNOT00000000212 | Forward +430 | 5'-GAGACACATCATGCTGGACACTTC-3' | 103.79% |
|  |  | Reverse +496 | 5'-AATAGAACTTTCGTCCAGGGGG-3' |  |
| Fasn | ENSRNOT00000073321.2 | Forward +1747 | 5'-CTTTGTGAGCCTCACCGCCAT-3' | 103.70% |
|  |  | Reverse +1811 | 5'-ATGCCATCAGGTTTCAGCCCC-3' |  |
| Gpam | ENSRNOT00000092170.1 | Forward +651 | 5'-AACTGAGACGAATCTGCCG-3' | 98.54% |
|  |  | Reverse +728 | 5'-AGGATGAAGGTGAGCAGCA-3' |  |
| Hmgcr | ENSRNOT00000022055.4 | Forward +679 | 5'-CATTCTTCCCAGCCTGCGTGT-3' | 102.28% |
|  |  | Reverse +745 | 5'-ATTGGACGACCCTCACGGCTT-3' |  |
| Hsd17b3 | ENSRNOT00000025850 | Forward +247 | 5'-CGGACACTGGAAAAGCTA-3' | 91.87% |
|  |  | Reverse +318 | 5'-TACAACCTTCACACGGCT-3' |  |
| Insig1 | ENSRNOT00000009233.6 | Forward +350 | 5'-TAGCCACCATCTTCTCCT-3' | 105.05% |
|  |  | Reverse +447 | 5'-CAGGTGACTGTCAATACAGG-3' |  |
| Ldlr | ENSRNOT00000013496.3 | Forward +823 | 5'-CAAGGACAAGTCGGACGAGGAGA-3' | 108.87% |
|  |  | Reverse +909 | 5'-CCGTGAATACAGGAGCCATCTGC-3' |  |
| Lxra | ENSRNOE00000127993 | Forward +311 | 5'-GCAGTGTATGTGGGGACAAGGC-3' | 100.17% |
|  |  | Reverse +399 | 5'-GATGACACTGCGGCGGAAGA-3' |  |
| Mttp | ENSRNOT00000014631.6 | Froward +1042 | 5'-TAGAACCTGAGAACCTGTCCAACGC-3' | 98.64% |
|  |  | Reverse +1113 | 5'-AAGTGCGGAGGTGCTGAATGAAG-3' |  |
| Npc1 | ENSRNOT00000016167.7 | Forward +1634 | 5'-CTTTGTCTATGCCGATTACC-3' | 91.16% |
|  |  | Reverse +1713 | 5'- GGAGCAAACTCGTATCATTC-3' |  |
| Rpl7a | ENSRNOT00000044551.4 | Forward +64 | 5'-GAGGCCAAAAAGGTGGTCAATCC-3' | 100.36% |
|  |  | Reverse +127 | 5'-CCTGCCCAATGCCGAAGTTCT-3' |  |
| Scap | ENSRNOT00000028295.7 | Forward +498 | 5'-ACCACAAACAAGGAGAGC-3' | 100.73% |
|  |  | Reverse +570 | 5'-ATCTGCTGGATGTATGCC-3' |  |
| Scarb1 | ENSRNOT00000064276.3 | Forward +555 | 5'-TTCGTTTCCAGCCAGACA-3' | 101.37% |
|  |  | Reverse +653 | 5'-GCTTGTCCTCCATCATCACT-3' |  |
| Scd | ENSRNOT00000018447 | Forward +942 | 5'-TCAATCTCGGGAGAACATCCTG-3' | 93.72% |
|  |  | Reverse +1013 | 5'-AAGGCGTGATGGTAGTTGTGGA-3' |  |
| Scp2 | ENSRNOT00000015420.5 | Forward +203 | 5'-GGCTATGTGTACGGTGAATCCA-3' | 99.33% |
|  |  | Reverse +280 | 5'-AATGATAGGGATGCCAGTCAGC-3' |  |
| Srebf1 | ENSRNOT00000047053.6 | Forward +613 | 5'-ACTGCTGTAAAGATGTACCCGTCCG-3' | 101.80% |
|  |  | Reverse +683 | 5'-GGCACTGGCTCCTCTTTGATTCC-3' |  |
| Srebf2 | ENSRNOT00000056041.2 | Forward +1048 | 5'-TCCCCATTAAGCAAGTGCCTGG-3' | 103.11% |
|  |  | Reverse +1117 | 5'-GTTGTCCGCCTCTCTCCTTCTTTG-3' |  |
| Star | ENSRNOT00000020606 | Forward +686 | 5'-CTTTGGGGAGATGCCTGAGC-3' | 98.89% |
|  |  | Reverse +765 | 5'-CAGCCAGTGGATGAAGCACC-3' |  |
| Tspo | ENSRNOT00000014089.4 | Forward +245 | 5'-ATCTGGGGCACACTGTATTC-3' | 82.12% |
|  |  | Reverse +330 | 5'-ACCATAGCCTCCTCTGTGAA-3' |  |

**Supplementary Table 1.** mRNA primers. Position refers to bp from the TSS of each gene. The qPCR amplification efficiency is calculated based on the slope of the standard curve. Slopes between -3.1 and -3.6 that have reaction efficiencies between 90 and 110% are typically acceptable. The efficiency calculator can be found online at:

https://www.lifetechnologies.com/us/en/home/brands/thermo-scientific/molecular-biology/molecular-biology-learning-center/molecular-biology-resource-library/thermo-scientific-web-tools/qpcr-efficiency-calculator.html.

| **Supplementary Table 2. MSP Primers** | | |  |
| --- | --- | --- | --- |
| Position | Methylation | Primers (5' → 3') | Efficiency |
| Srebf1 (ENSRNOT00000047053.6) | | | |
| Forward -127 | U | GTTTGGGTATTTGTAGGAGGTGGT | 91.09% |
| Reverse -29 | U | TAAACCCCACCCACCAACA |  |
| Forward -122 | M | GGTATTCGTAGGAGGTGGCG | 90.60% |
| Reverse -29 | U | TAAACCCCACCCACCAACA |  |
| Forward -127 | U | GTTTGGGTATTTGTAGGAGGTGGT | 88.02% |
| Reverse -36 | M | CGCCCACCAACGAAAATC |  |
| Forward +112 | U | GGAATAGGTATTGGTTGAGGTGTGT | 97.30% |
| Reverse +209 | U | ACCCAACACAAACCACACCA |  |
| Forward +119 | M | GGTATTGGTCGAGGTGTGCG | 97.31% |
| Reverse +209 | U | ACCCAACACAAACCACACCA |  |
| Forward +295 | U | TGGGATTGTAGTGGGTTTGGT | 85.57% |
| Reverse +386 | U | ACCCCAACTCAACCTATAAACTATACAC |  |
| Forward +295 | M | CGGGATCGTAGTGGGTTTG | 104.53% |
| Reverse +386 | U | ACCCCAACTCAACCTATAAACTATACAC |  |
| Forward +295 | U | TGGGATTGTAGTGGGTTTGGT | 120.24% |
| Reverse +382 | M | CGACTCGACCTATAAACTATACACAAAA |  |
| Srebf2 (ENSRNOT00000056041.2) | | | |
| Forward -537 | U | TGATTTAAGAATGTGTTAAGTTTTGGATG | 101.45% |
| Reverse -393 | U | CAAACACATTACATCATCTCCCCA |  |
| Forward -537 | U | TGATTTAAGAATGTGTTAAGTTTTGGATG | 94.91% |
| Reverse -393 | M | CGAACACATTACGTCATCTCCC |  |
| Forward -136 | U | TGTTTTGATTGGTTAATGTAGGTTTGG | 106.90% |
| Reverse -78 | U | CCTCCACACCCCCATATTTATTATC |  |
| Forward -136 | M | CGTTTCGATTGGTTAATGTAGGTTT | 92.21% |
| Reverse -78 | U | CCTCCACACCCCCATATTTATTATC |  |
| Forward -136 | U | TGTTTTGATTGGTTAATGTAGGTTTGG | 96.90% |
| Reverse -82 | M | CGCGCCCCCATATTTATTATC |  |
| Forward +366 | U | ATTGGGTTGTTGGTGAGGTTTT | 87.04% |
| Reverse +461 | U | CAACACCAAAACTCCTCAACCA |  |
| Forward +368 | M | CGGGTTGTCGGTGAGGTTT | 104.14% |
| Reverse +461 | U | CAACACCAAAACTCCTCAACCA |  |
| Forward +366 | U | ATTGGGTTGTTGGTGAGGTTTT | 104.67% |
| Reverse +461 | M | CGACGCCAAAACTCCTCAA |  |

**Supplementary Table 2.** MSP primers. Position refers to bp from the TSS of each gene. Primers are either methylated (M) or unmethylated (U). Slopes between -3.1 and -3.6 that have reaction efficiencies between 90 and 110% are typically acceptable. The qPCR amplification efficiency is given for primer pairs and is calculated based on the slope of the standard curve. The efficiency calculator can be found online at:

https://www.lifetechnologies.com/us/en/home/brands/thermo-scientific/molecular-biology/molecular-biology-learning-center/molecular-biology-resource-library/thermo-scientific-web-tools/qpcr-efficiency-calculator.html.
